# Dimensionality reduction and recurrence analysis reveal hidden structures of striatal pathological states

**DOI:** 10.3389/fnsys.2022.975989

**Published:** 2022-12-01

**Authors:** Miguel Serrano-Reyes, Jesús Esteban Pérez-Ortega, Brisa García-Vilchis, Antonio Laville, Aidán Ortega, Elvira Galarraga, Jose Bargas

**Affiliations:** ^1^División de Neurociencias, Instituto de Fisiología Celular, Universidad Nacional Autónoma de México, Mexico City, Mexico; ^2^Departamento de Ingeniería en Sistemas Biomédicos, Centro de Ingeniería Avanzada, Facultad de Ingeniería, Universidad Nacional Autónoma de México, Mexico City, Mexico; ^3^Department of Biological Sciences, Columbia University, New York, NY, United States

**Keywords:** neuronal ensembles, striatum, Parkinson’s disease, L-DOPA induced dyskinesia, UMAP, recurrence analysis, neural microcircuits, graph theory

## Abstract

A pipeline is proposed here to describe different features to study brain microcircuits on a histological scale using multi-scale analyses, including the uniform manifold approximation and projection (UMAP) dimensional reduction technique and modularity algorithm to identify neuronal ensembles, Runs tests to show significant ensembles activation, graph theory to show trajectories between ensembles, and recurrence analyses to describe how regular or chaotic ensembles dynamics are. The data set includes *ex-vivo* NMDA-activated striatal tissue in control conditions as well as experimental models of disease states: decorticated, dopamine depleted, and L-DOPA-induced dyskinetic rodent samples. The goal was to separate neuronal ensembles that have correlated activity patterns. The pipeline allows for the demonstration of differences between disease states in a brain slice. First, the ensembles were projected in distinctive locations in the UMAP space. Second, graphs revealed functional connectivity between neurons comprising neuronal ensembles. Third, the Runs test detected significant peaks of coactivity within neuronal ensembles. Fourth, significant peaks of coactivity were used to show activity transitions between ensembles, revealing recurrent temporal sequences between them. Fifth, recurrence analysis shows how deterministic, chaotic, or recurrent these circuits are. We found that all revealed circuits had recurrent activity except for the decorticated circuits, which tended to be divergent and chaotic. The Parkinsonian circuits exhibit fewer transitions, becoming rigid and deterministic, exhibiting a predominant temporal sequence that disrupts transitions found in the controls, thus resembling the clinical signs of rigidity and paucity of movements. Dyskinetic circuits display a higher recurrence rate between neuronal ensembles transitions, paralleling clinical findings: enhancement in involuntary movements. These findings confirm that looking at neuronal circuits at the histological scale, recording dozens of neurons simultaneously, can show clear differences between control and diseased striatal states: “fingerprints” of the disease states. Therefore, the present analysis is coherent with previous ones of striatal disease states, showing that data obtained from the tissue are robust. At the same time, it adds heuristic ways to interpret circuitry activity in different states.

## 1 Introduction

**FIGURE 1:**
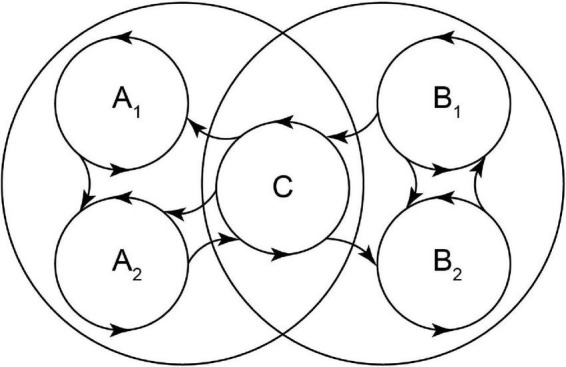
An illustration from Hebb that shows how recurrent transitions between neuronal ensembles might be (represented as “systems” in Hebb’s postulate). It proposes the possibility that ensemble C can serve as a link between two ensemble chains, thus participating in both, being the neuronal substrate for associative learning (Hebb, 1949). In addition, a modular theory can be inferred: each ensemble has inputs and outputs and performs a task step. Alternating activity between them performs the complete task or routine. Alternative pathways are possible to link different procedures. Tools described in the present article can show that this idea can be demonstrated with identified neuronal ensembles (cf., Figure 5).

The basic mechanisms of brain functions like perception, memory, attention, motor programs, emotions, and decision-making are now being studied using a variety of experimental techniques and theoretical frameworks (Rolls, 2016). Different pieces of knowledge are determined from each experimental/theoretical configuration. Realizing what objective data each one produces and putting them all together into a coherent “big picture” are both difficult tasks. Recently developed technologies for numerous simultaneous recordings and the computing power to evaluate them have led to a controversy over approaches that attempt to comprehend multicellular recordings and neuronal populations without sacrificing or omitting single cell resolution. The discovery that brain neurons do not act alone but rather collaborate to form groupings known as neuronal ensembles, which exhibit spatiotemporal coactivation, is significant (Yuste, 2015; Lara-González et al., 2022). When neurons in an ensemble are engaged in spontaneous, stimulated, diseased, or task-related activity, they fire in a coordinated manner (Ikegaya et al., 2004; Harris et al., 2011; Pérez-Ortega et al., 2016; Hamm et al., 2017; Sheng et al., 2019; Siniscalchi et al., 2019). The neural networks with emergent populational features have connections made between neuron groups rather than between individual neurons (Figure 1; Hopfield, 1982; Buzsáki, 2010; Kampa et al., 2011; Semedo et al., 2019; Rossi-Pool et al., 2021).

**FIGURE 2:**
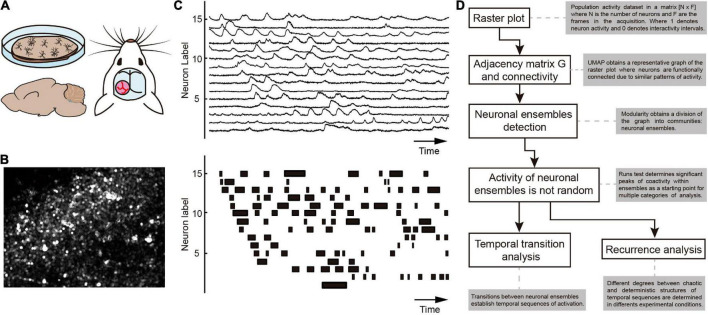
Analysis of calcium imaging experiments. **(A)** Calcium imaging movies can be acquired from neuron cultures (top left), brain slices (bottom left) or *in vivo* (right). With the aid of a molecule that fluoresces by binding calcium flowing into the activated neurons, the objective is to examine neurons that are present in the field of view. **(B)** An illustration of a video frame taken in a region of the brain where the fluorescent protein GCaMP6f is expressed in the cells. Regions of interest (ROI) are created from image sequences like this one to collect calcium signals. **(C)** Top: An illustration of calcium signals extracted from ROIs. Graphed as a raster plot at the bottom, neuronal activity inferred from calcium signals where each row represents the activity of a single neuron. Several techniques exist for creating raster plots from neural activity (Theis et al., 2016). **(D)** Once a raster plot has been created, an analysis pipeline is proposed and further described in the present work.

**FIGURE 3:**
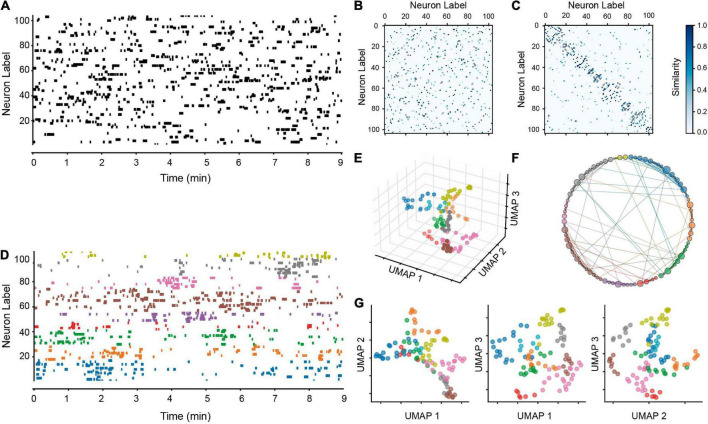
Identification of neuronal ensembles from calcium imaging experiments. **(A)** Raster plot. Each row corresponds to the activity of a neuron during an experiment. Black periods indicate moments of inferred electrical neuronal activity (events) where neurons have a high probability of firing action potentials. Dozens of neurons can be monitored simultaneously for several minutes, allowing the analysis of phenomena that can only be recorded in neuronal populations. **(B)** Adjacency matrix of neuronal activity (*V_A_*) determined with UMAP, neurons that are close together in the high-dimensional space display a functional connection (Fröhlich, 2016). **(C)** Adjacency matrix sorted after identifying neuronal communities: neurons show a significant number of connections within the same ensemble and significantly fewer connections with neurons from other ensembles. **(D)** Sorted raster plot after reordering the neurons according to their communities. These communities exhibit the properties attributed to neuronal ensembles (Buzsáki, 2010). Notice clusters of neurons (colored) alternating their activity following temporal sequences. **(E)** Low-dimension UMAP projection of active neuronal matrices identified in the experiment. Colors denote the same ensembles present in panel **(D)**. Neuronal ensembles occupy different positions in space after dimensional reduction. **(F)** Circular visualization of neuronal ensembles and their functional connections is obtained from the adjacency matrix B and colored as in panel **(D)**. Neurons present most of their connections with neurons of their own ensemble, and links among ensembles may represent the connectivity that enables the alternating activity during temporal sequences: how an ensemble is turned off when another is turned on. **(G)** Bidimensional projections in low dimension UMAP space to better appreciate communities’ separation from different angles.

**FIGURE 4:**
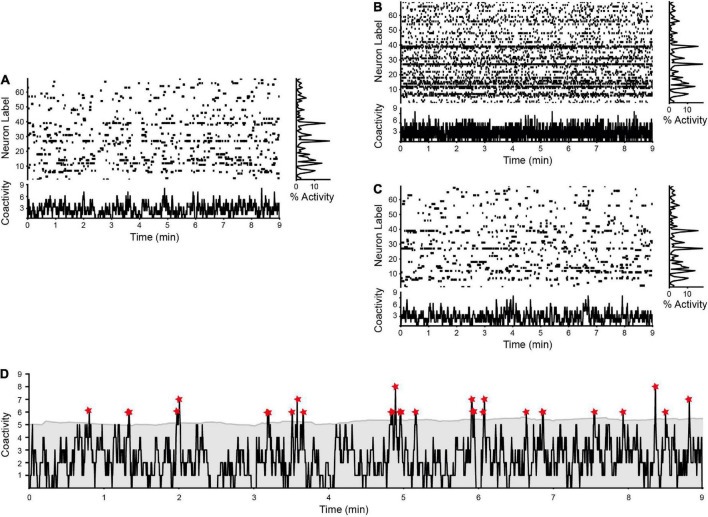
Determining significant coactivity peaks. **(A)** Raster plot and histogram of coactivity. Neuronal activity exhibits a unique space-time structure. **(B)** Plot of a representative surrogate neuronal raster obtained by maintaining the number of neurons, time, and number of firings of each one, but randomly permuting their activity on the *x*-axis. The coactivity histogram (bottom) shows a different pattern with respect to that observed in experimental data. However, note that the activity graph (at right) remains intact, revealing that the same level of neuronal activity is present. This class of shuffled raster plots the probability of executing a type 1 error as evaluated with a corresponding Runs test. **(C)** Another class of surrogate raster plot maintains neuronal activity with the same restrictions as in panel **(B)**, but with the same distribution of intervals between active moments after the random permutation. The pattern of coactivity determined through this process is like the one observed in the experiment. This yields a surrogate raster that is hard to distinguish from the experimental one. This surrogate raster plot is used to evaluate type 2 error with a corresponding Runs test. **(D)** Once shown that the experimental coactivity signal is not a product of chance, the significant values of coactivity are determined with a sliding window equal to “*n*” standard deviations (regularly *n* ≥ 2). A dynamic threshold is used to capture significant coactivity peaks. Asterisks indicate the time periods when the coactivity exceeds the threshold value.

**FIGURE 5:**
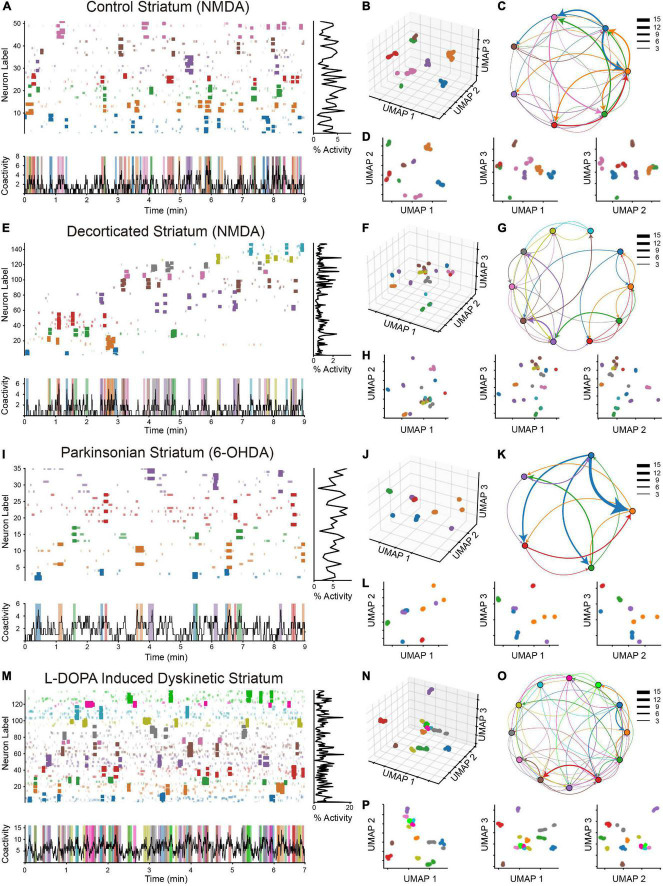
Dynamics of neuronal ensembles in different experimental conditions. **(A)** Significant activity of neuronal ensembles was identified in a raster plot obtained in the striatum under the control condition in the presence of N-methyl-D-aspartate (NMDA) to pharmacologically evoke the activation of network components; this brain nucleus is commonly very silent when there is no stimulus (spontaneous firing: Lara-González et al., 2019). The coactivation peaks are shown by darker colors, while the coactivation peaks that are not significant are indicated by paler colors for the same ensembles of neurons. **(B)** A low-dimensional UMAP projection of the significant peaks of coactivity (*V*_*P*_) that identify the significant coactive matrices from the raster plot. The colors denote identified neuronal ensembles, each in a specific niche in the UMAP space, confirming differences in the activation patterns of these neuronal groups (separation with the same color denotes the same neurons with different activation patterns). **(C)** Directed graph of neuronal ensemble temporal transitions; each node represents an ensemble. Arrows Edges are functional connections between them: color indicates the origin and arrowheads the destiny; the thickness of the edges represents the number of times a transition was carried out in the observed data. Some transitions can be identified in the raster plot even if the sorting procedure (see the Section 3.4) did not show them together. **(D)** Projections on the combinations of UMAP planes of the column vectors shown in section B. **(E–H)** Ensemble coactivation patterns in the decorticated striatum. Background neuronal activity is considerably lower than in control conditions and compared with the pathological states below (cf. histograms of % cell activity at right). Although numerous groups can be separated in section F, scarce temporal sequences (arrows in G) form a ring structure with many unidirectional connections. **(I–L)** Coactivation patterns of neuronal ensembles identified in striatal tissue depleted of dopamine (6-OHDA Parkinsonian model). There are fewer connections **(K)** between ensembles that are activated at regular times **(I)**. A more frequent transition appears (K; blue to orange). **(M–O)** Patterns of significant coactivation of neuronal ensembles identified in the striatum under the model of L-DOPA induced dyskinesia (LID): ensembles are projected in the low-dimensional space **(N)**, as well as transitions **(M,P)**, with several recurrences and high background activity (% activity histogram at right; note scale change).

Hebb (1949) proposed the neuronal ensembles theory as cell assemblies, and many research teams have recently refined it to propose them as the fundamental nervous system processing units (Figure 1; Buzsáki, 2010; Carrillo-Reid, 2021; Grillner, 2020, 2021; Lara-González et al., 2022). Although they may be referred to by different names, they commonly share several characteristics (Carrillo-Reid, 2021) and can be identified depending on context, i.e., responding together to stimulus (e.g., sensory inputs), causing an output (e.g., behavior), or representing experimental conditions (e.g., pathological states). It is considered that emergent properties of neural networks are a consequence of the spatiotemporal dynamics of coactive sets of neurons that alternate their activity following recurrent sequences that resemble computational routines or algorithmic processes that, together with a “modular” brain theory and analytical techniques, may generate a large-scale understanding of brain functions (Buzsáki, 2010). Here we expose a pipeline that may serve to ask whether neuronal samples of histological scale (dozens of neurons), commonly used for medical diagnosis, are representative of underlying ensembles of a larger scale by showing instructive and heuristic changes under different experimental conditions.

One multicellular recording technique that makes it possible to observe neuronal ensembles in culture, brain slices, or *in vivo* brain preparations from different animal models (Carrillo-Reid et al., 2008, 2009, 2016; Ahrens et al., 2013; Lock et al., 2015; Pérez-Ortega et al., 2016, 2021; Serrano-Reyes et al., 2020) is calcium imaging by using fluorescent indicators of neuronal activity, including the expression of genetically engineered calcium sensors in clusters of neurons *via* viral transfections or from birth using transgenic animals (Lin and Schnitzer, 2016; Wang et al., 2019). The purpose is to obtain videos of neuronal activity with single cell resolution in different experimental contexts (Yang and Yuste, 2017). The proposed pipeline uses the information from a video acquired through a calcium imaging experiment. However, it could be adapted to other multicellular recording techniques. Figure 2 depicts several calcium imaging setups as well as the method for locating regions of interest (neurons) and computing the fluorescence signals indicative of intracellular calcium levels. The final objective is to obtain the spatiotemporal information of the neuronal activity present in the field of view that can go from the histological to the mesoscale level, including thousands of neurons (Stringer et al., 2021). Instead of using “raw” calcium signals, which may show misleading relationships when evaluated as simultaneous recordings, spike timings estimated from calcium signals should be employed (Carrillo-Reid et al., 2008; Pérez-Ortega et al., 2016; Theis et al., 2016; Serrano-Reyes et al., 2020). Once this is done, analytical methods for identifying and studying neuronal ensembles are diverse, and a consensus has not yet been reached (Wenzel and Hamm, 2021). Different methods have been used in our laboratory with consistent and qualitatively similar outcomes, including hierarchical clustering, dimensionality reduction, similarity indices, and correlated activity (e.g., Carrillo-Reid et al., 2008, 2009, 2011; Jáidar et al., 2010; Pérez-Ortega et al., 2016; Serrano-Reyes et al., 2020; Duhne et al., 2021), indicating that, despite the constraints of calcium imaging, ensemble identification and activity sequences may provide robust data. But dimensionality reduction techniques are getting better all the time. Recent applications of the uniform manifold approximation and projection (UMAP) have been used in several contexts (e.g., Becht et al., 2019). Combined with modularity algorithms (Newman, 2006), it may become an optimal technique to identify communities in a population, called neuronal ensembles in the present context. Numerous techniques have identified the Parkinsonian circuit as characterized by exhibiting neuronal hyperactivity in the striatum as compared to the control, accompanied by a highly recurrent ensemble that greatly monopolizes the microcircuit at histological scale: the circuit gets stuck by this ensemble, as a “metaphor” of what happens with patients that cannot move or have difficulties doing it (Jáidar et al., 2010, 2019; Plata et al., 2013; Pérez-Ortega et al., 2016; Lara-González et al., 2019). Perhaps other disease states may also show a “fingerprint” when observed at the ensemble level (Fornito et al., 2016). The proposed pipeline allows us to describe disease by analyzing neuronal populations in *ex-vivo* brain slices.

## 2 Materials and equipment

The current work utilizes the database created by earlier laboratory work, which contains all information regarding experimental protocols and experimental subjects (Pérez-Ortega et al., 2016). A total of 37 *ex-vivo* brain slices from different mice were utilized in *n = 12* control, *11* decorticated, *7* parkinsonian, and *7* dyskinetic experiments. The movies were captured at a rate of 4 frames per second. The derivative criteria was applied to identify the periods of neuronal activity. The code in the python language and instructions to follow the analysis pipeline can be found at.^1^ Throughout this work, we use the UMAP v.0.5 Python implementation (McInnes et al., 2018),^2^ Brain Connectivity Toolbox for Python v.0.5.2 (Rubinov and Sporns, 2010),^3^ and the PyRQA tool to perform recurrence analysis in a massively parallel manner using the OpenCL framework (Rawald et al., 2017).

## 3 Methods

### 3.1 General considerations

Neuronal activity can be stored in a variety of ways; practical data structures rely on the temporal resolution and recording period (Paninski and Cunningham, 2018). The acquisition rates in calcium imaging experiments that last several minutes enable the construction of a brain activity monitoring matrix. There is previous work comparing actual with inferred neuronal activity from calcium imaging (Pérez-Ortega et al., 2016; Theis et al., 2016; García-Vilchis et al., 2018; Serrano-Reyes et al., 2020; Calderón et al., 2022). This matrix, usually called a raster plot (*R*), is a binary matrix of [*N* × *F*], where the *y*-axis denotes the number of neurons, *N*, whose individual activities comprise the rows (row vectors along the *x*-axis), and the *x*-axis represents the number of movie frames, *F*, which shows neurons that activate simultaneously at any given time (column vectors along the *y*-axis). Thus, *R* contains all the information necessary to study the population behavior through the simultaneous recordings of several neurons. It serves as the basis for figuring out the sequences of neuronal ensembles that alternate their activity during the experiment.

### 3.2 Determining the representative graph of neuronal activity using the uniform manifold approximation and projection

Uniform manifold approximation and projection is a dimensionality reduction algorithm that assumes that data samples are uniformly distributed in a topological high dimensional space. It learns the data manifold and then projects it into a lower dimensional space. UMAP accomplishes this goal through two main processes. First, it builds a graph connecting the nearest neighbors of each data point; this is achieved by choosing the distances between the points across the manifold, assuming they are uniformly distributed and connected to at least one other point. The next step for UMAP is to project or map the graph to a lower dimensional space. In this space, it is sought that the distances in the manifold do not vary with respect to the global coordinate system. Once this is achieved, the algorithm can start looking for a good low-dimensional representation by minimizing a cost function (Cross-Entropy) whose goal is to find the optimal weights of the connections. When this is finished, an array of the coordinates of each point in the specified data sample is depicted in a space of lower dimension, keeping the original structure as similar as possible (McInnes et al., 2018). In the raster plot *R* there are two vectors that may be prone to this dimensionality reduction: those in rows [1 × *F*] that represent the activity of individual neurons over time (*V_A_*) and those in columns [*N* × 1] that represent the coactive population at each instant of the experiment (*V_P_*). To identify and visualize neuronal ensembles and their temporal sequences of activation under different experimental conditions, we combine the UMAP methodology and graph theory algorithms on both the vectors of neuronal activity (*V_A_*, Figure 3) and the vectors of coactivation (*V_P_*, subsequently Figures 4, 5). Vectors of neuronal activity (*V_A_*) help to identify groups of neurons that coactivate with similar spatiotemporal patterns (Pérez-Ortega et al., 2016), that is, neurons that belong to the different neuronal ensembles that can be identified in each experiment (Figure 3A; Yuste, 2015).

The first step is to build a weighted graph, *G*, where each edge represents the probability that two nodes are functionally connected in our high-dimensional manifold (Fröhlich, 2016; McInnes et al., 2018). To determine connectivity, each *V_A_* is considered a sample from a continuous high-dimensional subspace (topology of neural activity). UMAP extends radiuses from each point to connect them by choosing the nth nearest neighbor. The connection of each node with its neighbors allows the local structure of the nodes to be maintained when performing subsequent manipulations (McInnes et al., 2018).

How “near” a particular point is to another is shown by the strength of each connection in the weighted graph. Since each point in this diagram represents a neuron’s vector of activity (*V_A_*; Figure 3A), the fact that two points are “near” indicates that their activity patterns are comparable. The result is the adjacency matrix *G* of size [*N* × *N*] that represents the weighted graph of the experiment. The next step is to determine a division of the graph into communities that reveals the existence of neuronal groups with similar activity patterns, that is, neuronal ensembles (Fröhlich, 2016; McInnes et al., 2018). The UMAP parameters are briefly discussed below in order to do this.

### 3.3 Description of uniform manifold approximation and projection parameters

The UMAP algorithm parameters are used to control the balance between local and global structure in the final projection of the data. The first parameter described is the approximate number of nearest neighbors (*N*_*NEIGHBORS*), which is used to construct the initial high-dimensional graph. It restricts the size of the local neighborhood that UMAP will look at when trying to learn the manifold structure of the data. Low values of this parameter will force UMAP to focus more on local structure by restricting the number of neighboring points considered when analyzing high-dimensional data. Small values should be used to capture fine details in the structure of neuronal activity (Becht et al., 2019) because it controls how UMAP balances the global vs. local structure of the data, ensuring fine granularity in building the ensembles. One concern is if this method can “dissolve” the high recurring ensemble discovered during parkinsonism by utilizing other clustering methods. If this occurs, what are the dynamics of the individual components? Do they follow specific types of temporal sequences? Another parameter is the *METRIC*, which controls how distances in multi-dimensional space are calculated from the input data. UMAP supports a wide variety of metrics, including Euclidean, normalized, angular, those used for binary data, and those based on paired correlations. In agreement with the original ideas of neuronal ensembles (Hebbian correlated activity), we use the metric based on correlations. In this way, the neurons that have functional connections represent those with strongly correlated activity patterns. The next parameter is the minimum distance between points in the low-dimensional space (*MIN*_*DIST*), which controls how tight the points are in an identified cluster. Larger values of *MIN*_*DIST* pack points more loosely, while smaller values lead to tighter packs. For finer clustering, the algorithm is favored by small values of this parameter (Becht et al., 2019). Essentially, the minimum spread of points can be controlled, thus avoiding scenarios with many points located on top of each other in the lower dimensional embedding. The default value *MIN*_*DIST* = 0.1, recommended in the UMAP documentation (see text footnote 2), is used in this paper. The last parameter to consider is *N*_*COMPONENTS*, which allows the user to determine the dimensionality of the reduced space where the data will be embedded to allow visualization. The most frequent numbers are 2 or 3, which correspond to the traditional dimensions that are simple to perceive. To generate the weighted graph, *N*_*NEIGHBORS* and *METRIC* are the most critical parameters, whereas *MIN*_*DIST* and *N*_*COMPONENTS* are crucial for producing the projection onto the low-dimensional UMAP space (McInnes et al., 2018). UMAP is one of the best tools for displaying highly dimensional data, and its success may be attributed to two very significant qualities: greater global structure preservation and more intelligible parameters. A crucial point to bear in mind is that anybody using the pipeline presented here should adjust the parameters (if necessary) in accordance with the characteristics of their data and their goals.

### 3.4 Identification of neural ensembles by using modularity and graphs to cluster neuronal activity

The relationships between the neurons (nodes) in the raster plot are depicted in the weighted graph *G* (see Section 3.2). The objective is to identify neuronal ensembles: groups of neurons that coactivate with comparable patterns of activity. Graph theory offers community extraction procedures to locate areas of the graph where groups of neurons (nodes) are highly coupled. The “modularity” algorithm (Newman, 2006) is one mechanism that maximizes the number of connections between elements that are in the same group and minimizes the number of connections between other groups. Modularity positive values indicate the possible existence of a community structure in the graph. Conversely, negative values indicate that a graph cannot be efficiently divided into communities.

The algorithm starts by calculating the modularity matrix *B* of a graph. Which is defined as:


$$B_{ij}=A_{ij}-\frac{k_{i}k_{j}}{2m}$$

Where the values *A*_*ij*_ are the elements of the adjacency matrix *G* determined previously with the help of UMAP (Figure 3B), *k_i_* and *k_j_* are the degrees of the nodes (i.e., their number of connections) and $m=\frac{1}{2}\sum_{i}k_{i}$ is the total number of connections in the graph. Identifying the biggest eigenvalue and determining its corresponding eigenvector comes after this matrix B has been calculated. In accordance with the sign of the eigenvector’s component elements, the graph is then split in half. The process is repeated for each of the parts using the formulation of the generalized modularity matrix *B*^(*g*)^:


$$B_{ij}^{\left(g\right)}=B_{ij}-\delta_{ij}\sum_{k\in g}B_{ik}$$

Where δ_*ij*_ is the Kronecker δ-symbol and $B_{ij}^{\left(g\right)}$ is an array [*n*_*g*_ *x* *n*_*g*_] with elements indexed by the labels *i*, *j* of vertices within a group *g*. At each stage, the contribution to the total modularity △*Q* is calculated through the following equation:


$$△Q=\frac{1}{2}\left(\frac{1}{2}\sum_{i,j\in g}B_{ij}\left(s_{i}s_{j}+1\right)-\sum_{i,j\in g}B_{ij}\right)$$

Where, for a particular division of the graph into two clusters let *s_i_ = 1* if vertex *i* belongs to group *1* and *s*_*i*_ = −1 if it belongs to group *2*. If at any stage of the algorithm, a proposed division is found to make a null or negative contribution to total modularity, the corresponding subgraph remains undivided. Numerous clustering proposals are gathered through this technique. To select a particular version, Bruno et al. (2015) iterate over the various clusterings until the same outcomes are found in most of them, producing a “consensus algorithm.” The algorithm finishes when it reaches this stage. There are a variety of iterations of the original method depending on how the beginning circumstances of the network division are put forth and how the modularity function is maximized (Newman, 2006).

Going back to the pipeline, Figure 3B displays the adjacency matrix *G* of the original raster (Figure 3A). The identical adjacency matrix *G* is shown in Figure 3C, but the nodes have been rearranged in accordance with the neuronal ensembles that have been identified and the interconnection that exists between them. Thus, Figure 3D displays a raster plot of the same neural activity as Figure 3A, but neuronal ensembles are shown from bottom to top according to the order in which they first appeared. Each ensemble is then given a color, and the sorted raster plot shows the temporal order. The color scheme utilized in the raster plot is retained in Figure 3E, which shows the projection of the activity vectors *V_A_* determined using UMAP in a three-dimensional UMAP space. The ensembles are separated from one another in the UMAP space, and the ensembles’ neurons are close to one another. Another method of visualizing the connections between neurons is to create a functional connectivity graph in a circular representation by arranging the colored-labeled neurons in the Figure 3D in a circle. The neurons of the same ensemble are grouped together in Figure 3F, and the size of the nodes corresponds to their number of connections. To see more clearly Figure 3E, projections of the vectors *V_A_* in the various planes are displayed in Figure 3G. The results show that UMAP identifies more ensembles than previous clustering methods (see below).

### 3.5 Determination of neuronal ensembles’ significant coactivity peaks (V_P_)

Once neuronal ensembles have been identified, it is necessary to show the transitions between them. As shown in Figure 3D, ensembles initiate, reach a peak, and wane. It is necessary to identify the precise instant at which they take part in a temporal ensemble sequence. Figure 4A top, shows an experimental raster plot obtained under control conditions in the striatum (Pérez-Ortega et al., 2016). At the bottom, a plot of neuronal coactivity is illustrated. It is a time series which consists of the sum of active neurons over time. To capture the occurrences in which a particular ensemble participates in ensemble sequences, periods in which neurons in a raster plot *R* exhibit significant peaks in coactivation should be identified. Time instants are the frames of the video, △*t*, each with a picture of the tissue at a certain time *t_n_*, which corresponds to the columns of the raster plot *R* (*V_P_*). It must be demonstrated that the significant coactivation peaks observed experimentally were not produced randomly. Here, we demonstrate how the “Runs test” —a non-parametric hypothesis test based on the binomial distribution— may be used to determine if a set of data can be explained by a random process (Bradley, 1968). The alternative hypothesis asserts that these values are not the result of a random process, while the null hypothesis claims that coactivity values randomly grow and decrease in accordance with a binomial distribution (Bujang and Sapri, 2018). Considering the two categories of statistical error: type 1 error (rejecting the null hypothesis when it is true) and type 2 error (failing to recognize significance when it is present). The significance level of the test, which is commonly set at α < 0.05, denotes the likelihood of making a type 1 error (for a discussion of this threshold, see Cowles and Davis, 1982; Glantz, 2012; Martinez and Martinez, 2015).

Runs test is important in determining whether a trial outcome is random for subsequent analysis (Bujang and Sapri, 2018) using the sorted neuronal ensemble subrasters (set of vectors *V_A_* belonging to neuronal ensembles) for further analysis. The first step in the Runs test is to count the number of runs in the data sequence. A “run” is defined as a series of consecutive increasing or decreasing values with respect to the mean, where the duration of the run is given by the number of these values. In a random data set, the probability that the (*i* + 1)-th value is greater than or less than the *i*-th value follows a binomial distribution. The statistic is:


$$Z=\frac{T-\bar{T}}{s_{T}}$$

Where *T* is the observed number of runs, $\bar{T}$ is the expected number of runs, and *s_T_* is the standard deviation of the number of runs. The values of $\bar{T}$ and *s_T_* are calculated as:


$$\bar{T}=\frac{2n_{1}n_{2}}{n1+n2}+1$$

$$s_{T}^{2}=\frac{2n_{1}n_{2}\left(2n_{1}n_{2}-n_{1}-n_{2}\right)}{{\left(n_{1}+n_{2}\right)}^{2}\left(n_{1}+n_{2}-1\right)}$$

**FIGURE 6:**
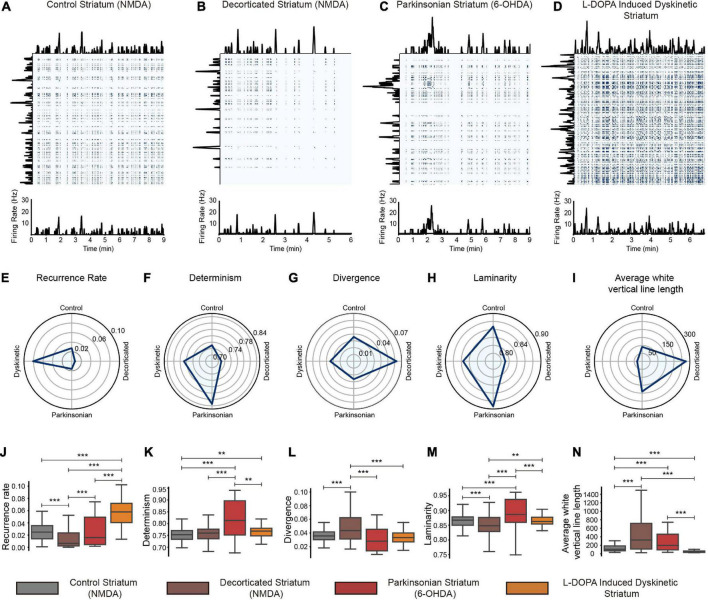
Recurrence quantification analysis of the firing rate of representative neuronal ensembles. **(A–D)** Top: recurrence plots of the firing rate of neuronal ensembles of the striatum in the different conditions. At the left and upper ends of the matrices, the traces being analyzed are observed, and for better appreciation, they are also shown at the bottom. Note that, except for matrix B, all conditions exhibit lattice structures, differing in their spacing. **(E)** The recurrence is higher for the dyskinetic state. **(F)** The determinism is higher for the parkinsonian condition, suggesting predictability. **(G)** The divergence is higher for the decorticated condition, suggesting a more chaotic state. **(H)** The laminarity score is for the parkinsonian circuits, the most regular. **(I)** The average white vertical line length is higher for the decorticated state, where recurrent times are the longest–almost isolated ensembles. The last row **(J–N)** shows the comparisons between the samples determined in the complete dataset and essentially confirms what was observed in the representative example quantifications shown in the upper row **(E–I)**. The actual values can be read in the main text.


Where *n_1_* and *n_2_* denote the number of positive and negative values in the series. The resultant score is compared to the normally distributed, two-tailed confidence interval. It is determined that the alternative hypothesis is correct when the value of the experimental series is higher than that attained by a random series (Mendenhall and Reinmuth, 1982). To apply this method to the data, a dichotomous time series made up of the subrasters coactivity values (*V_A_*), where values above the mean are positive and values below the mean are negative, is build. In the case of the Runs test applied to the coactivity of the experimental raster plot of Figure 4A (this test is later applied to Figures 5, 6), a value of *Z* = 30.05 was determined, which corresponds to *p* < 0.0001, allowing the null hypothesis to be rejected: the coactivity time series is not a result of chance. Surrogate matrices (Figures 4B,C) must be created while maintaining the same number of neurons, time, acquisition rate, and active frames for each neuron to demonstrate that neither type 1 nor type 2 errors exist. To evaluate the type 1 error, these surrogate matrices are generated by placing in a uniform distribution the instants of activation of each of the neurons during the period of the experiment. Knowing that the null hypothesis should not be rejected, the outcome of test type 1 error should be noted as:

$$I_{i}=\{\begin{array}{c} 1\\ 0 \end{array}\begin{array}{c} TypeIerrorismade\\ TypeIerrorisnotmade \end{array}$$

The procedure for determining *I_i_* is repeated for *M* (1,000) surrogates and the probability to make a type I error is calculated as:


$$\hat{\alpha }=\frac{1}{M}\sum_{i=1}^{M}I_{i}$$

This value is an estimate of the significance level of the test for a given critical value. Applied with an exemplary surrogate matrix (e.g., Figure 4B), a value of $\hat{\alpha }<0.05$is determined, suggesting there is not a type 1 error.

To estimate type 2 error, surrogate matrices must not satisfy the null hypothesis, which requires that the raster matrices not be random (e.g., Figure 4C). Consequently, surrogate matrices now conserve the parameters used in the type 1 error test adding the interactivity intervals of each neuron (equivalent to interspike intervals in electrophysiological recordings). With these restrictions, a pseudo-population of neuronal activity like the one observed is determined. The hypothesis test is performed with this surrogate raster matrix, and the value *I_i_* is recorded. In the case of a type 2 error, this value is produced as:


$$I_{i}=\{\begin{array}{c} 1\\ 0 \end{array}\begin{array}{c} TypeIIerrorismade\\ TypeIIerrorisnotmade \end{array}$$

The probability of making the type 2 error after *M* (1,000) surrogates is:


$$\hat{\beta }=\frac{1}{M}\sum_{i=1}^{M}I_{i}$$

In the case described a value of $\hat{\beta }$ < 0.0001 is determined, suggesting there is not a type 2 error. Be aware that while the formulae are similar, the surrogate matrix type varies. Once it is determined that our experimental coactivity time series is not randomly generated, its upper extreme values are extracted: the “peaks of coactivity” that can be interpreted as marks in time when column vectors (*V_P_*) had significant coactivation of neurons during the experiment.

A sliding window is constructed in the coactivity time series and moved one by one to the right until it reaches the end of our data set in order to achieve these values. This window is built using a value that is expressed as a percentage of the data from the time series’ beginning to end. Typical values include 5, 10, or 20%. For each window, the mean and standard deviation are calculated to yield a local threshold. If it is greater than the mean + two standard deviations, then point is considered to be a significant peak of coactivity. Figure 4D shows the result of applying sliding windows of size 20% to the coactivity plot of Figure 4A. An asterisk is placed at each time where the real value of coactivity exceeds the variable threshold. With this procedure, significant peaks of coactivation (*V_P_*) are determined from the subrasters (set of *V_A_* vectors). Different thresholds can be used with little change in the outcome.

### 3.6 Characterizing the activity of neuronal ensembles *via* recurrence analysis

Recurrence analysis was used to conduct a more quantitative assessment of the ensemble neuronal transition dynamics. This approach allows us to quantify the frequency and length of the neuronal ensemble recurrences. Instead of the suggestions that were previously employed (Carrillo-Reid et al., 2009; Pérez-Ortega et al., 2016; Lara-González et al., 2019; Duhne et al., 2021; Calderón et al., 2022), it presents a new potential of interpretations. In light of this, it is possible to model the behavior of neuronal ensembles as a dynamic system. A dynamical system evolves over time within a state space according to a given rule. This evolution is represented in “phase space.” The state of a system in time *t* is specified by a point in phase space, and the progression of the system generates an orbit or trajectory through this space. Using recurrence plots, it is possible to demonstrate how neuronal ensembles identified using the methods previously described (see above) behave over time in each diseased condition, leaving a “signature” (Argyris et al., 2015). A recurrence plot is a non-linear data analysis technique that allows visualizing those times in which a state of a dynamic system is repeated, revealing all the moments in which the trajectory of the phase space of a dynamic system visits the same area in the phase space. To build a recurrence analysis, let $\vec{x_{i}}$ be the *i*-th point on the orbit describing a dynamical system in *d*-dimensional space, for *i* = 1, *N*. The recurrence plot *P* is a matrix of dots in a [*N* × *N*] square, where a dot is placed at (*i*, *j*) whenever $\vec{x_{j}}$ is sufficiently close to $\vec{x_{i}}$ (Packard et al., 1980; Eckmann et al., 1987).


Pi,j={1:0:    xi→xi→   ≈≈   xj→xj→   i,j=1,… ,N

In this case, $\vec{x_{i}}\vec{x_{j}}$indicates the similarity of a pair of vectors. The matrix *P* captures a total of *N*^2^ binary similarity values. A distance measure is needed to determine the similarity between pairs of vectors. There are several alternatives (Manhattan, Euclidean, maximum distance; Webber and Zbilut, 1994). Here, the Euclidean distance is utilized for simplicity. A neighborhood condition is applied to transform the pairwise similarities into binary values. In the fixed radius condition, the binary values are determined by a threshold ∈. All vectors that lie within the ∈-neighborhood of a query vector $\vec{x_{q}}$ are considered like $\vec{x_{q}}$ (Poincaré, 1890). A strategy is to choose an ∈ threshold based on the density of the recurrence plot (Zbilut et al., 2002). A fixed radius value of ∈ = 1.5 was applied to the data for the current proposal. All the parameters described below were determined from objects in the RQAComputation class of the PyRQA tool (library mentioned in Section 2). The values of the fixed radius (neighborhood requirement) and the metric to measure the similarity between the vectors must be supplied in order to generate such an object (for the implementation see the script titled Figure 6 at see text footnote 1). Statistical comparisons used Mann–Whitney tests with Holm–Sidak *post-hoc* adjustment.

The parameters that recurrence quantification analysis extracts from recurrence matrices *P* are: (1) The recurrence point density, or recurrence rate (*RR*), that is defined as:


$$RR=\frac{1}{N^{2}}\sum_{i,j=1}^{N}P_{i,j}$$

Where *P*_*i, j*_ are the entries of the recurrence matrix *P*. This parameter quantifies the proportion of recurrence points that are determined with a specified radius. When *N* →, ∞ *RR* is the probability that a state recurs to its ∈-neighborhood in phase space. (2) Another measure is based on diagonal lines; it is called determinism (*DET*). It refers to the portion of recurrence points that form diagonal lines. Only diagonal lines with a length of *l* ≥ 2 are considered regarding the quantitative analysis:


$$DET=\frac{\sum_{l=2}^{N}lP_{D}\left(l\right)}{\sum_{i,j=1}^{N}p_{i,j}}$$

Where *lP*_*D*_(*l*) is the number of points of the recurrence matrix that form diagonal lines of size *l*. Chaotic signals (aperiodic and presenting sensitivity to initial conditions) yield short diagonal lines, periodic or deterministic signals yield long diagonal lines, and stochastic signals do not show diagonal lines (Webber and Zbilut, 1994). *DET* parameter is a measure of the order, predictability, or rigidity of the system. (3) Divergence (*DIV*) is the inverse of the length of the longest diagonal line found in the recurrence plot:


$$DIV=\frac{1}{max\left({\left\{l_{i}\right\}}_{i=1}^{N_{l}}\right)}$$

Where *N_l_* is the total number of diagonal lines. This parameter is conjectured to be related to the Lyapunov exponent, which estimates the rate at which signal paths diverge. Thus, for larger *DIV* values, a time series is more chaotic (Eckmann et al., 1987), (4) Laminarity (*LAM*) captures the number of recurring points that form vertical lines. The equation to determine this parameter is identical to the equation to determine *DET*, with the exception that, in this instance, the length of the vertical lines is measured rather than the length of the diagonal lines. *LAM* will decrease if the recurrence plot consists of more single recurrence points than vertical structures. This parameter allows us to investigate how a signal evolves over time through the concept of intermittency. A signal is called intermittent if its temporal evolution appears to be regular for long periods of time and is interrupted from time to time by brief irregular intervals with amplitudes of greater intensity. Intermittency, in this regard, indicates a seamless change from regular periodic behavior to chaotic behavior (Marwan et al., 2007; Argyris et al., 2015). (5) The length of continuous vertical lines formed by values with *P*_*i*, *j*_ = 0 (average “white” vertical line length *W*). The length of these lines is equal to the time needed by the system to recur to a previously visited state in such a way that it serves as an estimator for recurrence times (Ngamga et al., 2007).

## 4 Results

### 4.1 Vectors of neural activity (V_A_) are used to determine neuronal ensembles

Hebbian theory (Figure 1; as used by previous authors: Eichenbaum and Davies, 1998) served as the foundation for the proposed pipeline (Figure 2) for determining neuronal ensembles. The analysis of raster plots showing inferred neuronal activity from calcium imaging experiments performed on brain slices was done using the differentiation method (Pérez-Ortega et al., 2016).

The first stage in the process (Figure 2D) is to use UMAP to recognize the neuronal ensembles from a raster plot (Figure 3A). This enables the creation of a representative experiment adjacency matrix (Figure 3B). The ideal split of the matrix into communities that theoretically correspond to the neural ensembles is then determined using the addition of the modularity method (Figure 3C). The detected neural ensembles are then displayed in the original raster plot after it has been sorted (Figure 3D; colored). Every ensemble or collection of neurons creates a time series with recurrence. The same colors used in the sorted raster display are used in Figure 3E to project these neuron groups into a low-dimensional environment. The neurons are then arranged in a circle-shaped representation, with each neuron acting as a node and each edge representing a connection between neurons (Figure 3F). Two-dimensional projections are also provided as a different way to visualize the division of neuron groups (Figure 3G).

Each neuronal ensemble is put through the Runs tests after being divided into its own time series to prove that its activation patterns are not the result of chance (Figure 4; see the Section 3.5). Then, using this data, two analytical procedures are carried out: (1) to examine the significant activation patterns of the sequences of each neuronal ensemble (see below), and (2) to find out whether the activity rates of each neuronal ensemble recur (see below).

### 4.2 The activation pattern of the significant coactivity peaks (V_P_) characterizes the pathological conditions in the striatum

In order to highlight potential research directions that address concerns regarding neuronal population dynamics, Figure 5’s left side presents four raster plots (Figures 5A,E,I,M) that were obtained in the striatum under different experimental conditions (Pérez-Ortega et al., 2016). The neuronal ensembles in these striatal microcircuits were determined using previously described methods and are rendered in different colors. A more pronounced hue is used to highlight the significant *V_P_* vectors of each neuronal ensemble that were identified *via* the Runs tests ($\hat{\alpha }$ < 0.05; $\hat{\beta }$ < 0.0001; in all analyzed raster plots; see the Section 3.5 and Figure 4). Note that not all activity within an ensemble belongs to significant peaks of coactivity, but only precise moments with a low probability of appearing at random are considered. The transitions between the significant coactivity peaks, which occur when one ensemble stops being active and another one starts, define the temporal sequences between neuronal ensembles. The coactivity histogram at the bottom of the raster plots displays colored vertical bars that indicate the intervals in which each neuronal ensemble has a significant *V_P_* vector. Each colored vertical vector appears recurrently throughout the course of time, and sequences between different vectors can also be viewed repeatedly (e.g., blue-orange-green-red in Figure 5A). The middle column of Figure 5 shows the projection of these significant coactivation vectors in the low-dimensional UMAP space (*R*^3^; Figures 5B,F,J,N; and for a better visualization in different planes: Figures 5D,H,L,P). These projections represent the states of the system and behave as attractors of neuronal activity because, by definition, transitions between them produce trajectories that recur once and again. The right column illustrates graphs showing these state transitions (arrows) and trajectories (cf. Figure 1 showing alternative trajectories), where each node represents a neuronal ensemble (not a neuron as in previous work: Pérez-Ortega et al., 2016; Duhne et al., 2021; Calderón et al., 2022; Figures 5C,G,K,O). Each directed arrow increases its thickness in agreement with the number of times a transition occurs between a given pair of ensembles involved. For better rendering and tracking, the edge color is the same as the origin node, and the destination node is indicated by the arrowhead. Accordingly, directionality indicates the presence of temporal transitions between neuronal ensembles and forms a way of representing the flexibility of a system to codify computations, given that these state transitions with several alternative trajectories (Figure 1) have historically been associated with underlying mechanisms of brain functions (Hebb, 1949; Buzsáki, 2010; Carrillo-Reid, 2021; Lara-González et al., 2022). Next, each experimental condition is described.

The control conditions’ raster plot (with NMDA in the bath since the striatum has low spontaneous activity; Carrillo-Reid et al., 2008; Lara-González et al., 2019) displays seven distinct ensembles (Figure 5A), which are nearly fully segregated from one another in low-dimensional space (Figure 5B) and exhibit alternating activity (denoted by arrows in the state transition graph) that forms temporal sequences and several state transitions throughout time (Figure 5C). Analyzing different control networks in different slices (*n = 12*), number of ensembles was (mean ± SEM): 7.33 ± 0.51 and the number of transitions: 28.17 ± 2.25. Note that hierarchical clustering showed only three ensembles using the same data (Pérez-Ortega et al., 2016), suggesting that ensembles determined with previous clustering methods can be further subdivided with UMAP and modularity clustering. Background activity around the significant peaks of coactivity is seen as more opaque color, and the whole activity density may be approximated by the histogram of % cellular activity at right. Figure 5B shows the projection of the significant peaks of coactivity (*V_P_*) in *R*^3^ and Figure 5D shows the different combinations of *R*^2^ planes that can be formed. Each ensemble takes up a specific area in space; the more similar the vectors are, the closer their projections are to one another. Therefore, it may be inferred that some neuronal ensembles are carrying out computations for a specific purpose when their projections are brought closer together in space. On the other hand, it may be assumed that a neuronal ensemble is performing different actions if projections for a given set are seen in several locations (note that this is always inside a manifold). It appears that information processing is stable under the control situation since neuronal ensembles are seen in highly differentiated locations. In the control condition, many temporal sequences can be found, present among the ensembles with some predominant ones, some of which can be observed directly in the raster plot (e.g., blue-orange). The proportion of the number of transitions is balanced. Although some transitions are more recurrent, there is not a predominant sequence in the microcircuit.

Next, in the decorticated striatum (Figure 5E; in the presence of NMDA), which is an experimental condition where the striatum has been deprived of the cortical inputs that survive after the slicing procedure (Arias-García et al., 2018; Aparicio-Juárez et al., 2019). Note that, again, several groups are projected in the low-dimensional space (Figure 5F). Analyzing different decorticated networks in different slices (*n = 11*), ensembles were 9.18 ± 0.84, and the number of transitions was 33.91 ± 3.63, not significantly different from the controls. Interestingly, some neuronal ensembles have different states denoted by the same color and separated in low-dimensional space. The phase transition graph (Figure 5G) shows a “ring” structure with many transitions in the same direction, a signature of regular networks (Watts and Strogatz, 1998). This behavior may be characteristic of the striatum in the absence of inputs “commanding” the circuit into its “standard” mode, as proposed in other brain regions (Raichle and Snyder, 2007). For example, background activity is clearly diminished (cf. histogram of % cellular activity at right; notice different scales), suggesting that a cortical driving force is needed for striatal background activity (Garcia-Munoz et al., 2015) and the generation of ensemble temporal sequences (Carrillo-Reid et al., 2008; Lara-González et al., 2019).

Figure 5I shows the raster plot and coactivity histogram for the Parkinsonian condition. For dopamine depletion, the 6-OHDA model of hemiparkinsonism was used (Jáidar et al., 2010). Spontaneous activity in the dopamine depleted striatum has been quantified and demonstrated to be augmented with respect to the control condition without NMDA (Lara-González et al., 2019). Therefore, NMDA was not added in this condition. Projections of neuronal ensembles in low-dimensional space (Figure 5J) are shown. One query is if the highly recurrent ensemble that manifests in this pathological model (Jáidar et al., 2010) can be “dissolved” by UMAP and modularity clustering, and if so, whether detected sub-ensembles alternate their activity or, conversely, display recurrent behavior impeding a balanced alternation. The results show that indeed, significant peaks of coactivity projected in the low-dimensional space show several neuronal ensembles separated by their similar firing patterns (Figure 5J). Analyzing different parkinsonian networks in different slices (*n = 7*), the ensemble number was 5.71 ± 0.52 significantly different from the decorticated and dyskinetic conditions (*p* = 0.03 and *p* = 0.05, respectively; Mann–Whitney with Holm–Sidak *post-hoc* tests), and the number of transitions was also significantly reduced: 14.71 ± 2.34 (*p* = 0.01 vs. all other conditions; Mann–Whitney with Holm–Sidak *post-hoc* tests). Therefore, the temporal structure of the raster plot is different to the control: first, there are fewer transitions between ensembles (Figure 5K), second, a dominant recurrent sequence appears on the network (Figure 5K blue-orange), third, although arrows denoting auto-recurrence on the same ensemble are not depicted, some ensembles in the raster plot (Figure 5I) can be observed to have regular times of appearance (e.g., orange, green, and red). In contrast, the raster plot’s activity density is higher than the decorticated striatum and similar to the control (see the histograms of cellular activity to the right). Therefore, despite revealing more neuronal ensembles, the ensemble identification algorithm confirms previous results formerly quantified with other metrics (Lara-González et al., 2019): the dopamine depleted striatum acquires a highly repetitive structure.

Finally, what is revealed by this neuronal ensemble detection algorithm from L-DOPA induced dyskinetic striatal tissue (*n = 7*; Figure 5M; Winkler et al., 2002) is described. The projection of significant peaks of coactivity on the low-dimensional space shows various neuronal ensembles: 8.71 ± 0.81 (Figure 5N) and transitions between them: 38.43 ± 4.36 (Figure 5O) with less silent periods (vertical colored lines on the coactivity histogram in Figure 5M), perhaps underlying the hyperkinesia and involuntary movements present in this condition. All of this is accompanied by an increased firing density as a background activity (cf. histograms at right showing % of cellular activity; note different scales).

In summary, a simpler and statistically consistent detection of significant peaks of coactivity is coherent with previous methods using shuffled data and MonteCarlo simulations: both alternating activation and temporal sequences of neuronal ensembles are detected, with multiple alternative pathways as previously proposed (Figure 1). However, UMAP and modularity analysis find more neuronal ensembles by dissolving the highly recurrent ensemble previously found in the striatal pathological states using the same database (Jáidar et al., 2010; Pérez-Ortega et al., 2016; Calderón et al., 2022), but leaving important functional connections between ensembles as seen with phase transition graphs. Whether these findings confirm or alter the prior understanding of striatal diseased states provided by other dimension reduction and clustering approaches is the key question. We next go on to the recurrence analysis to wrap off our investigation of this subject.

### 4.3 Recurrence analysis of neuronal ensembles in control and pathological states

Recurrence analysis uses activity rates over time to build recurrence plots (Figure 6). For each experimental condition, activity rates with a 1-s sliding window of representative neuronal ensembles were taken from the same database. The first row of Figure 6 shows the recurrence plots with the time series insets corresponding to the firing rates used to calculate them. The middle row has the quantifications of the recurrence analysis parameters for each recurrence plot of the representative ensembles shown in the top row. The last row of Figure 6 shows the comparisons of these parameters in the neuronal ensembles determined from the complete dataset. Except panel 6B, all other panels in the upper row of Figure 6 have a lattice structure, the difference being the level of granularity. The Parkinsonian state (hypokinesia) has the largest empty spaces (Figure 6C), while the dyskinetic state has the smallest (hyperkinesia; Figure 6D), with control conditions in between (Figure 6A). The *DIV* parameter shows that the more chaotic structure is the decorticated striatum (Figure 6G for cases in the top row and Figure 6L for the entire sample), showing that even in a brain slice maintained *in vitro*, surviving corticostriatal afferents contribute to neural ensemble dynamics (Arbuthnott and Garcia-Munoz, 2017; Arias-García et al., 2018; Aparicio-Juárez et al., 2019). This validates both cortical stimulations to intervene in striatal dynamics during disease (Beuter et al., 2014) and *in vitro* techniques to evaluate these therapeutic procedures (Aparicio-Juárez et al., 2019). Recurrence analyses also show that the Parkinsonian state is dissimilar to the decorticated state, a fact that remained obscure with previous observations and analyses, namely, that the cortex helps in maintaining the Parkinsonian state.

The number of elements in each sample was *n*_*control*_ = 88, *n*_*decorticated*_ = 101, *n*_*parkinsonian*_ = 40 and *n*_*dyskinetic*_ = 61 neuronal ensembles obtained from *n = 12* control, *11* decorticated, *7* parkinsonian, and *7* dyskinetic experiments from *37 ex-vivo* slices of different mice. Neuronal ensembles were identified as explained in see the Section 3.4. The observed values are expressed as mean ± 2 standard errors of the mean (SEM) and are dimensionless. The results are the following:

The most recurrent state is that from dyskinetic tissue (Figures 6E,J) with a value of *RR*_*dyskinetic*_ = 0.056 ± 0.006 (*p* < 0.001 compared to all conditions), reflecting that this microcircuit maintains both types of recurrence: alternating activity between ensembles (*V_P_* vectors) and recurrent activity between the same ensembles (*V_A_* vectors; Figure 5D). The temporal sequences between ensembles are also more intricate. It is followed by the control condition value of RR_control_ = 0.027 ± 0.004 (Figures 5A, 6J), which, in addition to the significant difference with the dyskinetic state described above, shows a higher value than the decorticated condition (*p* < 0.001). The neuronal ensembles of the Parkinsonian state have a recurrence value like the control condition (RR_parkinsonian_ = 0.026 ± 0.008). But they lack the variety of transitions between the neuronal ensembles that the control condition exhibited (Figures 5C, 6J). Instead, the Parkinsonian microcircuits present a dominant recurrent transition with respect to the others. Control and Parkinsonian circuits show a higher significant value of recurrence with respect to the decorticated condition (*p* = 0.001). Finally, decorticated tissue exhibits a substantial difference: almost no recurrence (RR_decorticated_ = 0.016 ± 0.004, Figure 6J), its ensembles being almost isolated, showing another discrepancy with Parkinsonian circuits (Jáidar et al., 2010, 2019; Plata et al., 2013; Pérez-Ortega et al., 2016; Lara-González et al., 2019). Most of the values determined in the dataset are less than 0.1, confirming that the recurrence matrices are sparse as in other observed dynamical systems (Marwan et al., 2007).

The Parkinsonian state is the most deterministic, rigid, or predictable (DET_parkinsonian_ = 0.818 ± 0.025; *p* < 0.001 compared against the control and decorticated conditions; *p* = 0.01 compared against the dyskinetic condition; Figures 6F,K). Dyskinetic (DET_dyskinetic_ = 0.764 ± 0.006), decorticated (DET_decorticated_ = 0.757 ± 0.007), and control conditions (DET_control_ = 0.754 ± 0.007) have lower values in that order. The only significant difference is observed between the control and dyskinetic conditions (*p* = 0.02; Figure 6K). This result goes along with the paucity of transitions of the Parkinsonian state (Figure 5C) as compared to the others, as well as with the high recurrence of the same ensembles observed before (Jáidar et al., 2010). The circuit’s characteristics and its original metaphor—a highly recurrent and predictable circuit—were thus unaffected by the disappearance of the highly recurring diseased condition that was seen using other approaches. The distinction with the decorticated state is obvious. The control circuit does not fall between the Parkinsonian and dyskinetic circuits, according to this measure, which is interesting because it has a lower score than both. Perhaps this result is the consequence of the underlying Parkinsonian state during L-DOPA induced dyskinesia; a comparison with other choreic states (e.g., Huntingtonian circuit) is therefore necessary.

The decorticated state presents the highest divergence value (*DIV*_*decorticated*_ = 0.818 ± 0.025; *p* < 0.001 compared to other conditions; Figure 6L), indicating a greater tendency of this type of system to present irregular or chaotic behavior, i.e., the need for cortical inputs to generate direction in striatal actions (*DIV*_*control*_ = 0.037 ± 0.002; *DIV*_*parkinsonian*_ = 0.033 ± 0.006; *DIV*_*dyskinetic*_ = 0.034 ± 0.002). On the other hand, laminarity is analogous to determinism (stretch of vertical lines instead of diagonal lines) in such a way that a low value of it represents a system with many fluctuations. In this sense, the Parkinsonian circuit again shows the highest score compared to the other conditions. (*LAM*_*parkinsonian*_ = 0.888 ± 0.017; *p* < 0.01 in each comparison; Figures 6H,M). When a system presents high values of *DET* and *LAM*, it is highly likely that it has structures that are repeating themselves rigidly over time, in this case, the activity of their neuronal ensembles. However, there is a significant difference between both measures. Whereas *LAM* represents the probability that a specific value will not change over time, *DET* measures the probability that similar changes in the activity rate recur. The similarity between control and dyskinetic states using this metric emphasizes the lattice nature of both recurrence plots (*LAM*_*control*_ = 0.865 ± 0.005; *LAM*_*dyskinetic*_ = 0.863 ± 0.005). The decorticated circuit showed a significantly lower value of laminarity with respect to the other conditions (*LAM*_*decorticated*_ = 0.846 ± 0.009; *p*_*vs. control*_ = 0.005; *p*_*vs. dyskinetic*_ = 0.017).

Finally, the decorticated circuit has the longest average white vertical line length or recurrence times, which measures how long it takes for the system to return to a state that has already been visited (W_decorticated_ = 486.964 ± 96.460; Figure 6I). It is significantly larger than the control and dyskinetic conditions (*p* < 0.001 in both comparisons), but no differences were found with respect to the Parkinsonian state, emphasizing that ensembles found in this decorticated state are isolated (Pérez-Ortega et al., 2016). The Parkinsonian circuit (W_parkinsonian_ = 284.925 ± 71.624), explained by the paucity of transitions (Figures 5C, 6C,I), was the next highest (*p*_vs. control_ = 0.004; *p*_vs. dyskinetic_ < 0.001). With this metric, the control circuit stays between the dyskinetic and Parkinsonian circuits (W_control_ = 159.342 ± 41.827; W_dyskinetic_ = 57.001 ± 9.339; *p*_vs. parkinsonian_ = 0.004; *p*_vs. *dyskinetic*_ < 0.001). The above-described pipeline has the potential to differentiate experimental conditions.

## 5 Discussion

The present reinterpretation of striatal circuitry diseased states at histological scale (dozens of neurons) following the methodology for determining neuronal ensembles based on dimension reduction and graph theory plus recurrence analysis shows a novel viewpoint on how to represent these sophisticated systems. It is demonstrated that a larger number of neuronal ensembles are revealed compared to earlier clustering techniques (Carrillo-Reid et al., 2008; Pérez-Ortega et al., 2016). Once the UMAP settings have been configured to produce a representative graph from a raster display, the modularity technique does not require any parameters to identify the neuronal ensembles. As a result, greater transitions between them occur in the control and dyskinetic states as opposed to the decorticate or parkinsonian conditions. Transitions between ensembles are more evenly distributed in the control condition (in NMDA because in the control, “resting” or inactivated striatum, the neuronal activity is relatively scarce; Lara-González et al., 2019). Interestingly, recurrence is similar in the control and the Parkinsonian states. Therefore, a bulk measure of activity between the control activated striatum and the DA-depleted one may show no differences if measured with multi-recording techniques. The difference must be found in how this activity is structured. In fact, the measure of how long it takes for the system to return to a state that has already been visited (average white vertical lines) shows that the lattice structure of the control condition resides in between the Parkinsonian and the dyskinetic states.

### 5.1 Cortical afferents and diseased states

After the slicing technique, cortical afferents that remain in the parasagittal slices are crucial for sustaining control, parkinsonian, and dyskinetic states (Arias-García et al., 2018). In the decorticated condition, in addition to less background activity, it also presents almost isolated neuronal ensembles (Pérez-Ortega et al., 2016) with unidirectional transitions, the higher divergence (suggesting entrance into a chaotic state), the lowest recurrence rate, and the higher average white vertical line, denoting longer periods of time in which the system tends to recur. Likewise, the greater dispersion observed in this last parameter reflects its chaotic behavior. Together, these data support the evidence that minimal corticostriatal inputs are necessary to structure the striatal parkinsonian circuitry (Arbuthnott and Garcia-Munoz, 2017) and validate cortical stimulation to intervene in striatal diseased states (Beuter et al., 2014; Aparicio-Juárez et al., 2019). In conclusion, the striatal circuitry is not like a decorticated circuitry; it needs the cortex to be maintained, perhaps denoted by the role of the dominant β rhythm oscillation found in motor cortices in parkinsonian conditions (Little and Brown, 2014).

### 5.2 The parkinsonian state

While the background activity and the recurrence between the control and Parkinsonian circuits are similar, their low-dimensional structure is quite different. Parkinsonian circuits show a lower number of neuronal ensembles, fewer transitions between them, and predominant recurrent sequences. Graph theory methodology found more ensembles than with previous algorithms; however, they have more regular activation times. Recurrence analysis shows that the Parkinsonian state becomes more deterministic, rigid, or predictable and usually remains in laminar periods (less turbulent) in greater proportion than the other conditions. Therefore, even though the method of determining neuronal ensembles presented here “dissolves” the highly recurrent state found with alternative algorithms (Jáidar et al., 2010; Plata et al., 2013) into several sub-states, the network acquires highly recurrent sequences that interrupt alternation anyway. Consequently, a stringent clustering algorithm was put to test, but it did not contradict the basic “metaphor” of the histological level of analysis found with other algorithms: a deterministic network with highly recurrent sequences still mirrors what is observed in Parkinsonian patients: rigidity and a lack of ability for goal directed and postural control movements, or hypokinesia-akinesia. Finding similar conclusions with different algorithms shows that what is observed in the tissue in each condition are robust findings of data on neuronal populations. Therefore, analyses become complementary but, in this case, with greater statistical robustness.

### 5.3 The dyskinetic state

For the *ex-vivo* tissue in L-DOPA induced hyperkinetic conditions, the analysis confirmed previous reports with other methods (Pérez-Ortega et al., 2016; Calderón et al., 2022): neuronal ensembles are multiplied as well as transitions between them. Further supporting the “metaphor” of patients exhibiting an enhancement of stereotyped hyperkinetic involuntary movements along with the underlying Parkinsonian state, these findings show that more transitions between ensemble sequences are accompanied by recurrent sequences, with this network having the highest recurrence. This network needs to be compared to other choreic states. This condition also has the highest density of background activity, and it is the circuit with the shortest average white vertical line, reflecting the tight lattice structure of the recurrence matrix, suggesting that cortical inputs are facilitated (Guerra et al., 2019). In fact, certain parameters of cortical stimulation may aid in the treatment of L-DOPA-induced dyskinesia (Martini et al., 2019).

## 6 Concluding remarks

The method of extracting neuronal ensembles from calcium imaging experiments presented here proved to be capable of serving as a starting framework for computational analyses of neuronal populations. The results could be statistically substantiated. The pipeline presented may be implemented in other studies with relative ease. A powerful feature of the method is the possibility to reconstruct, in pieces (neuronal ensembles), the whole activity observed in a portion of brain tissue, allowing the study of neural states. Here, we demonstrate how recurrence analysis might be used to examine a dataset of recordings made from a particular brain area under several experimental setups. It was possible to differentiate between the diseased states and the controls and to reinterpret the data, showing new aspects of the cortico-striatal relations and the Parkinsonian and dyskinetic states. Note that cortical inputs’ absence is not an absence of inputs since some inputs coming from the thalamus (e.g., Arias-García et al., 2018) and from the external globus pallidus (e.g., Mallet et al., 2012) remain. In addition, the pipeline may be used to design neuronal ensembles based pre-clinical bioassays to evaluate potentially useful drugs to treat neurological ailments, trying to avoid entering the multiple biochemical signaling and biophysical details that single neuron recordings or whole tissue measurements imply (Plata et al., 2013; Lara-González et al., 2019; Barack and Krakauer, 2021; Calderón et al., 2022). The clinical stage may be the use of biopsies from surgery patients if the general picture or population analysis becomes a fingerprint of the disease. Therefore, the proposed pipeline of analysis becomes crucial for this line of research.

## Data availability statement

Publicly available datasets were analyzed in this study. This data can be found here: https://github.com/MiguelSerranoReyes/neuronal-ensembles.

## Ethics statement

The animal study was reviewed and approved by the Institutional Committee for Laboratory Animals Care and Use of the Instituto de Fisiología Celular (IFC), UNAM (NOM-062-Z00-1999; laboratory protocols JBD-59-15).

## Author contributions

MS-R and JB wrote the first draft of the manuscript. JP-O obtained the analyzed data. BG-V and AO coded and debugged the code used. AL performed the review of the mathematical methods. EG performed the review of experimental methods. All authors wrote sections of the manuscript, contributed to manuscript revision, and read and approved the submitted version.

## Footnotes

1
https://github.com/MiguelSerranoReyes/neuronal-ensembles

2
https://github.com/lmcinnes/umap

3
https://github.com/aestrivex/bctpy
